# Psychische Belastungen bei Erwerbstätigen: Leistungsinanspruchnahme und Kosten für das deutsche Gesundheitssystem

**DOI:** 10.1007/s00103-024-03901-w

**Published:** 2024-06-11

**Authors:** Nadine Mulfinger, Peter Angerer, Yesim Erim, Nicole Hander, Marieke Hansmann, Regina Herold, Reinhold Kilian, Christoph Kröger, Eva Rothermund, Jeannette Weber, friaa-Studiengruppe, Jolanda Brezinski, Manuel Feisst, Fiona Kohl, Meike Heming, Harald Gündel, Kristin Herrmann, Rike Seega, Sinja Hondong, Lorena Brenner, Sophia Chrysanthou, Volker Köllner, Ralf Stegmann, Ute B. Schröder, Uta Wegewitz, Tamara Waldmann

**Affiliations:** 1 Klinik für Psychiatrie und Psychotherapie II der Universität Ulm am Bezirkskrankenhaus Günzburg, Lindenallee 2, 89312 Günzburg, Deutschland; 2 Institut für Arbeits‑, Sozial- und Umweltmedizin, Centre for Health and Society, Medizinische Fakultät, Heinrich-Heine-Universität Düsseldorf, Düsseldorf, Deutschland; 3 Abteilung für Psychosomatische Medizin und Psychotherapie, Universitätsklinikum Erlangen, Friedrich-Alexander-Universität Erlangen-Nürnberg (FAU), Erlangen, Deutschland; 4 Klinik für Psychosomatische Medizin und Psychotherapie, Universitätsklinikum Ulm, Ulm, Deutschland; 5 Institut für Psychologie, Abteilung Klinische Psychologie und Psychotherapie, Universität Hildesheim, Hildesheim, Deutschland

**Keywords:** Inanspruchnahme von Gesundheits- und Sozialdiensten, Krankheitskosten, Arbeitsbezogene Psychotherapie, Rückkehr an den Arbeitsplatz, Gesundheitsökonomische Evaluation, Health and social services use, Medical expenses, Work-related psychotherapy, Return to work, Health economic evaluation

## Abstract

**Einleitung:**

Die Inanspruchnahme von Leistungen durch Arbeitnehmer:innen mit psychischen Belastungen sowie die damit verbundenen Kosten im Gesundheits- und Sozialsystem wurden bisher nicht systematisch in Studien erhoben bzw. nur indirekt erfasst. Diese Publikation hat zum Ziel, die Inanspruchnahme in dieser Zielgruppe zu dokumentieren, die Kosten im Gesundheits- und Sozialsystem erstmalig abzuschätzen und mögliche Einflussfaktoren der Kostenvarianz zu untersuchen.

**Methodik:**

Als Teil einer Multicenter-Studie wurden Häufigkeiten der Inanspruchnahme sowie Kosten im Gesundheits- und Sozialsystem von 550 Arbeitnehmer:innen mit psychischen Belastungen erhoben. Die Inanspruchnahme von Leistungen wurde mit der deutschen Version des Client Sociodemographic Service Receipt Inventory (CSSRI) erhoben. Kosten wurden für 6 Monate berechnet. Mithilfe eines Regressionsmodells wurden Einflussfaktoren auf die Kosten überprüft.

**Ergebnisse:**

Zu Studienbeginn betrugen die durchschnittlichen Gesamtkosten der vergangenen 6 Monate in der Stichprobe € 5227,12 (Standardabweichung € 7704,21). Das Regressionsmodell weist auf einen signifikanten Anstieg der Kosten mit zunehmendem Alter sowie bei Personen mit Depressionen, Verhaltensauffälligkeiten mit körperlichen Symptomen und anderen Diagnosen hin.

**Diskussion:**

Die berechneten Kosten sind im Vergleich zu klinischen Stichproben ähnlich hoch. Des Weiteren sollte zukünftig untersucht werden, ob sich dieses Ergebnis durch die Analyse der Längsschnittdaten verändert und ob die Intervention einen Kosteneinfluss aufweist.

## Abstract (en)

**Background:**

Service use among employees with mental health problems and the associated costs for the health and social system have not yet been systematically analysed in studies or have only been recorded indirectly. The aim of this article is to report the service use in this target group, to estimate the costs for the health and social system and to identify possible influencing factors on the cost variance.

**Methods:**

As part of a multicentre study, use and costs of health and social services were examined for a sample of 550 employees with mental health problems. Service use was recorded using the German version of the Client Sociodemographic Service Receipt Inventory (CSSRI). Costs were calculated for six months. A generalized linear regression model was used to examine influencing cost factors.

**Results:**

At the start of the study, the average total costs for the past six months in the sample were € 5227.12 per person (standard deviation € 7704.21). The regression model indicates significant associations between increasing costs with increasing age and for people with depression, behavioural syndromes with physiological symptoms, and other diagnoses.

**Discussion:**

The calculated costs were similar in comparison to clinical samples. It should be further examined in longitudinal studies whether this result changes through specific interventions.

## Einleitung

Laut DAK-Gesundheitsreport sind psychische Erkrankungen nach Atemwegs- und Muskel-Skelett-Erkrankungen der dritthäufigste Grund für krankheitsbedingte Arbeitsunfähigkeitszeiten. Im Jahr 2022 konnten 15 % der Krankenstandstage in Deutschland auf psychische Erkrankungen zurückgeführt werden [1]. Zugleich sind eine beeinträchtigte psychische Gesundheit oder bereits diagnostizierte psychische Erkrankungen eine der häufigsten Ursachen für Frühberentungen [2, 3]. Wissenschaftliche Erkenntnisse aus Berichten der Organisation für wirtschaftliche Zusammenarbeit und Entwicklung (Organisation for Economic Co-operation and Development, OECD) weisen darauf hin, dass etwa 15 % der Bevölkerung im erwerbsfähigen Alter unter „common mental disorders“ (CMD) oder leichten psychischen Beeinträchtigungen (z. B. leichte bis mittelgradige depressive Episoden oder Angststörungen) leiden [4].

Die beschriebenen Arbeitsunfähigkeitszeiten verursachen hohe volkswirtschaftliche Kosten. So entstanden im Jahr 2021 allein in Deutschland ca. 15,8 Mrd. € Produktionsausfallkosten und 27,1 Mrd. € Ausfall von Bruttowertschöpfungen aufgrund psychischer Belastungen [5]. In allen Mitgliedsstaaten der Europäischen Union kann laut einer OECD-Auswertung für das Jahr 2015 davon ausgegangen werden, dass psychische Erkrankungen direkte und indirekte Kosten von insgesamt 600 Mrd. € verursachen [6]. Eine verzögerte Inanspruchnahme von Hilfe, z. B. aufgrund fehlender wahrgenommener Notwendigkeit, kann überdies zu reduzierter Lebensqualität, Stigmatisierung und gesamtgesellschaftlichen Produktivitätsverlusten führen. Hinzu kommt, dass lediglich etwa 20 % der Menschen mit psychischen Belastungen eine angemessene Behandlung erhalten [7]. Früherkennung und Frühintervention bei psychischen Belastungen können hingegen die Prognose positiv beeinflussen [2, 8]. Erste wissenschaftliche Erkenntnisse zeigen, dass eine niedrigschwellige psychotherapeutische Intervention am Arbeitsplatz depressive Symptome reduzieren und die Arbeitsfähigkeit verbessern kann [9]. Insbesondere die Kombination aus Prävention, frühzeitiger Behandlung, arbeitsbezogener Psychotherapie und Zusammenarbeit zwischen den wichtigsten Fachkräften der (psychischen) Gesundheitsversorgung scheint vielversprechend, wenn es darum geht, eine Chronifizierung, lange und wiederholte krankheitsbedingte Arbeitsunfähigkeitszeiten sowie Frühverrentung aufgrund von CMD zu verhindern [1, 3]. Studien im beruflichen Umfeld untersuchten bisher hauptsächlich Interventionen, die eine Wirksamkeit in Bezug auf Symptome, Arbeitsfähigkeit [9, 10], Arbeitsunfähigkeitstage [10, 11] oder die Rückkehr an den Arbeitsplatz [10, 12] erwarten ließen. Studien der Kosteneffektivität von frühzeitigen Interventionen zu psychischer Gesundheit am Arbeitsplatz in Deutschland fehlen aber bisher.

Aus einer gesundheitsökonomischen Perspektive stellt sich bei der wissenschaftlichen Evaluation derartiger Maßnahmen insbesondere die Frage, ob diese zu einer Reduzierung der volkswirtschaftlichen Kosten psychischer Erkrankungen im beruflichen Umfeld beitragen. Dazu ist es zunächst notwendig, die mit dem Auftreten und der Behandlung psychischer Erkrankungen in Verbindung stehenden Krankheitskosten vollständig zu erfassen. Wichtig ist hierbei, dass die Kostenerfassung neben den durch die gesetzliche Krankenversicherung (GKV) gedeckten Kosten auch die Kosten anderer Sozialversicherungsträger wie der gesetzlichen Rentenversicherung (RV) erfasst. Des Weiteren müssen auch die mit einer psychischen Erkrankung verbundenen indirekten Kosten aufgrund von Produktivitätsverlusten berücksichtigt werden [13, 14].

In Deutschland wurden Krankheitskostenanalysen für psychische Erkrankungen bislang überwiegend für Populationen mit schweren psychischen Erkrankungen („severe mental illness“ [SMI]), wie z. B. Schizophrenie oder schweren depressiven Episoden, durchgeführt [15–18]. Trotz der deutlich höheren Prävalenz von CMD liegen zu den Kosten dieser Erkrankungen bislang kaum Untersuchungen vor. Grund hierfür ist die Fokussierung auf Behandlungssektoren, die hohe Kosten für die GKV verursachen, insbesondere die stationäre Krankenhausbehandlung. Ein weiterer Grund könnte darin liegen, dass Personen mit einer Erkrankung aus dem SMI-Spektrum durch entsprechende diagnostische Kriterien einfacher kategorisierbar und häufiger in spezifischen Behandlungssettings, wie z. B. psychiatrischen Kliniken, auffindbar sind. Dabei bleibt jedoch unberücksichtigt, dass CMD wegen ihrer besseren Behandlungsmöglichkeiten und ihrer großen Verbreitung größere Möglichkeiten einer volkswirtschaftlichen Einflussnahme bieten als häufig bereits chronifizierte und in ihrem Verlauf nur noch schwer beeinflussbare Formen von SMI.

Untersucht werden im Rahmen dieses Beitrags die Inanspruchnahme und die Kosten medizinischer und psychosozialer Gesundheitsleistungen einer Population mit Erkrankungen, die vielmehr den CMD zuzuordnen sind. Die Erreichbarkeit dieser Population über betriebliche Settings bildet dabei einen entscheidenden Vorteil gegenüber Studien mit primär klinischen Populationen. Die Ergebnisse der Untersuchung liefern Informationen zu Krankheitskosten in frühen Phasen der Entwicklung psychischer Störungen und bilden damit eine Grundlage für die gesundheitsökonomische Evaluation von primär- und sekundärpräventiven Interventionen.

Im Arbeitskontext auf europäischer Ebene konnten Wissenschaftler:innen aus Schweden zeigen, dass die Acceptance-and-Commitment-Therapie (ACT) für Personen mit Depressionen und Angsterkrankungen kosteneffektiv sein kann, aber nicht für Personen mit stressbedingter Erschöpfungssymptomatik [19]. Ein niederländisches Team untersuchte eine strukturierte Online-Selbsthilfeintervention für Personen mit depressiven Symptomen auf Kosteneffektivität und konnte diese, abhängig von der Höhe der Zahlungsbereitschaft von Entscheidungsträgern, bestätigen [20]. Detailliertes Wissen hinsichtlich kosteneffektiver Interventionen am Arbeitsplatz, die auf die Behandlung psychischer Erkrankungen und Belastungen abzielen, ist bisher eher rar.

Das Projekt „Frühe Intervention am Arbeitsplatz“ (friaa) zielt darauf ab, Arbeitnehmer:innen mit psychischen Belastungen möglichst früh durch eine niedrigschwellige arbeitsbezogene Psychotherapie am Arbeitsplatz zu unterstützen und somit die Wartezeiten im Gesundheitssystem zu verkürzen und die psychische Gesundheitsversorgung zu verbessern, Arbeitsunfähigkeitszeiten zu reduzieren bzw. das Ausscheiden aus dem Arbeitsleben zu verhindern [21].

Ein langfristiges Ziel im Rahmen von friaa ist es, mögliche Einsparpotenziale im Gesundheits- und Sozialsystem aufzuzeigen. Dafür müssen in einem ersten Schritt alle Gesundheits- und Sozialleistungen erfasst und bepreist werden. Des Weiteren soll geklärt werden, welche Faktoren Einfluss auf die Höhe der Kosten haben. Im vorliegenden Beitrag sollen daher alle Gesundheits- und Sozialleistungen ermittelt werden, die die Teilnehmenden im 6‑Monats-Zeitraum vor Beginn der Intervention in Anspruch genommen haben. Dieser Beitrag dient somit als Referenz für die während der Interventionsphase in Anspruch genommenen Leistungen und damit einhergehenden Kosten.

## Methodik

### Studiendesign

Als Teil einer Multicenter-Studie wurde von Arbeitnehmer:innen mit psychischen Belastungen die Häufigkeit der Inanspruchnahme von Leistungen sowie die daraus resultierenden Kosten im Gesundheits- und Sozialsystem erhoben. Die Teilnehmenden wurden im Zeitraum von September 2021 bis Januar 2023 in 5 deutschen Studienzentren (Düsseldorf, Erlangen, Hildesheim, Teltow, Ulm) aus Groß-, mittelständischen und Klein(st)unternehmen rekrutiert. Die Studie wurde zum einen intern in den Unternehmen (u. a. durch Betriebsärzt:innen oder Mailings) und zum anderen über lokale Zeitschriftenartikel und Social Media bekannt gemacht. Die Teilnahme an der Studie war freiwillig. Interessierte kontaktierten das Studienteam, wurden auf Erfüllung der Einschlusskriterien gescreent und anschließend zufällig der Interventions- oder Kontrollgruppe zugeteilt (randomisiert).

Einschlusskriterien waren eine schriftliche Studieneinwilligung, Volljährigkeit, für die Studienteilnahme ausreichende Deutschkenntnisse, eine Beschäftigung von mindestens 15 h pro Woche, eine diagnostizierte psychische Erkrankung (CMD) nach ICD-10 (F32–34, F42, F43, F45, F48, F51; erhoben anhand des Mini-International Neuropsychiatric Interview (M.I.N.I.); [22]) oder Symptome einer psychosomatischen Erkrankung ohne ICD-10-Diagnose, gemessen mit der Global-Assessment-of-Functioning-Skala (GAF) mit einem Wert von < 81 [23, 24].

Ausschlusskriterien waren ein diagnostizierter Substanzmittelmissbrauch (F10–19), Schizophrenie, Psychosen (F20–29) oder eine organische psychische Erkrankung (F00–09), schwere körperliche Erkrankungen (z. B. Krebs), gegenwärtige psychotherapeutische Behandlung oder ein laufender Antrag auf (Früh‑)Berentung.

### Intervention

Die Teilnehmenden der Interventionsgruppe nahmen an bis zu 17 Psychotherapieeinheiten teil. In den ersten beiden Sitzungen wurden diagnostische und medizinische Indikationsverfahren angewandt. Bis zu 10 weitere Sitzungen wurden mit dem Fokus einer arbeitsbezogenen Psychotherapie durchgeführt [25]. Bei Bedarf folgten im Anschluss bis zu 5 Sitzungen, falls eine Person arbeitsunfähig war und an den Arbeitsplatz zurückkehrte. Die Teilnehmenden der Kontrollgruppe erhielten die übliche Behandlung (Treatment as Usual [TAU]) sowie die erste Sitzung des diagnostischen Indikationsverfahrens. Die psychotherapeutischen Angebote wurden i. d. R. durch psychologische oder ärztliche Psychotherapeut:innen durchgeführt. Nähere Details zur Intervention finden sich im Studienprotokoll [21]. Studienbefragungen fanden vor der Randomisierung und Interventionsbeginn (Baseline, T0), nach 9 (T1) und 15 (T2) Monaten statt. Die vorliegende Auswertung basiert auf den Daten der Baseline-Befragung zum Messzeitpunkt T0 (September 2021 bis Januar 2023). Die Untersuchung erfasst die Inanspruchnahme und die Kosten von Gesundheitsleistungen sowie die indirekten Krankheitskosten aufgrund von Produktivitätsverlusten und erfüllt damit die Anforderungen einer Analyse aus der volkswirtschaftlichen Perspektive.

### Messinstrumente

Die Inanspruchnahme von Leistungen wurde mit der deutschen Version des Client Sociodemographic Service Receipt Inventory (CSSRI) erhoben [26, 27]. Die Beantwortung des Fragebogens dauerte durchschnittlich 10 min. Anhand des CSSRI wurden 8 Leistungskategorien erhoben: (1) stationäre und (2) tagesklinische Krankenhausaufenthalte der letzten 6 Monate, (3) ambulante ärztliche und (4) therapeutische Gesundheitsleistungen, wie z. B. Besuche beim/bei der Hausärzt:in oder dem/der Physiotherapeut:in, (5) Ambulanzen und Notdienste, (6) gesundheitsbezogene Beratungsstellen, wie z. B. Suchtberatung, (7) Onlinebehandlungen oder -beratung und (8) Medikamenteneinnahmen. Die Kategorien 3 bis 7 wurden für die vorangegangenen 3 Monate erhoben, Medikamente für den letzten Monat. Hinzu kamen Angaben zum Erwerbsstatus und der Wohnsituation. Jeder in Anspruch genommenen Leistung wurde der entsprechende Kostensatz zugewiesen, anschließend wurden alle Kosten mit dem Faktor 2 bzw. 6 multipliziert, um die Leistungsinanspruchnahme bzw. Kosten für 6 Monate auszuweisen.

Diagnosen wurden mittels M.I.N.I. [22] erhoben und anschließend gruppiert nach Depressionen (F32–33), Angststörungen (F40–41), Anpassungsstörungen (F43), somatoformen Störungen (F45), Verhaltensauffälligkeiten mit körperlichen Störungen (F50–59) sowie andere Diagnosen (Nominalskalierung). Des Weiteren wurde mithilfe des GAF [23, 24] beurteilt, inwiefern der/die Teilnehmende psychisch in der Lage ist, die sozialen oder beruflichen Funktionsbereiche auszufüllen. Die Skala reichte von 1 (ständige Gefahr, sich oder andere zu verletzen; Unfähigkeit, eigene Hygiene aufrechtzuerhalten) bis 100 (hervorragende Leistungsfähigkeit, keine Schwierigkeiten). Ab einem Wert von < 81 (vorübergehende Symptome bzw. Reaktionen auf psychosoziale Belastungsfaktoren) wurden die Teilnehmenden in die Studie aufgenommen.

### Kostenzuweisung

Die Kosten für die erfassten Leistungseinheiten wurden aus verschiedenen Quellen entnommen. Stationäre Kostenangaben entstammen dem Entgeltsystem Psychiatrie, Psychotherapie und Psychosomatik (PEPP; [28]), dem DRG-Entgelttarif [29] und Muntendorf et al. [30]. Ambulante Kostenangaben wurden dem Einheitlichen Bewertungsmaßstab (EBM; [31–34]) und dem Einheitlichen Bewertungsmaßstab für zahnärztliche Leistungen (BEMA; [35]) entnommen. Kosten des Sozialsystems wurden bei Trägern oder Dienstleistern ermittelt (Tab. 1). Kosten für Medikamente wurden dem aktuellsten deutschen Arzneiverordnungsreport von 2022 entnommen, in welchem die Kosten für definierte Tagesdosen hinterlegt sind [36]. Für Personen, die Arbeitsunfähigkeitstage angegeben hatten, wurden indirekte Kosten in Form von Produktionsausfallkosten berechnet. Der zugrunde gelegte Stundenlohn von € 24,63 wurde auf Basis des Durchschnittsbruttoeinkommens von 2022 errechnet [37].

**Tab. 1:** Einheitskostenliste

	Details	Einheit	Einheitskosten (in €)	Quelle	Jahr
Stationäre Krankenhausbehandlung	Psychiatrische Klinik	1 Tag	277,92	InEK GmbH [28]	2022
	Psychosomatische Klinik	1 Tag	277,92	InEK GmbH [28]	2022
	Somatische Klinik	1 Tag	1011,72	Muntendorf et al. [30]	2020
	Psychiatrische Rehabilitation	1 Tag	277,92	InEK GmbH [28]	2022
	Notaufnahme	Pro Fall	120,00	Mühlenfeld et al. [58]	2021
Tagesklinik	Psychiatrische Tagesklinik	1 Tag	208,44	InEK GmbH (0,75*277,92; [28])	2022
	Psychosomatische Tagesklinik	1 Tag	208,44	InEK GmbH (0,75*277,92; [28])	2022
	Somatische Tagesklinik	1 Tag	208,44	InEK GmbH (0,75*277,92; [28])	2022
Ambulante ärztliche Leistungen	Psychiater:in	Pro Besuch (10 min)	21,63	KBV, EBM, Quartal 2, S. 459 [32]	2022
	Arzt/Ärztin für psychosomatische Medizin	1 h	106,47	InEK GmbH [28]	2022
	Psychotherapeut:in und Psycholog:in	Pro Besuch (erste 10 min)	8,90	KBV, EBM, Quartal 1, S. 477 [32]	2022
	Psychotherapeut:in und Psycholog:in	Alle weiteren 10 min	17,35	KBV, EBM, Quartal 1, S. 479 [31]	2022
	Psychotherapeut:in und Psycholog:in	1 h	95,65	KBV, EBM, Quartal 1, S. 479 (8,90 + (5*17,35); [31])	2022
	Allgemeinmediziner:in	Pro Besuch	18,12	KBV, EBM, Quartal 2, S. 151 f. [32], Grundpauschale, Mittelwert der altersspezifischen Angaben	2022
	Andere Praktiker:innen	Pro Besuch	22,23	KBV, EBM, Quartal 2, S. 357 [32], Grundpauschale, Mittelwert der altersspezifischen Angaben	2022
	Zahnarzt/Zahnärztin	Pro Besuch	42,12	KZVLB, BEMA [35]	2023
	Ambulante psychiatrische Versorgung	Pro Tag	95,89	KBV, EBM, Quartal 1, S. 479 (8,90 + (5*17,35); [31])	2022
	Schlaflabor	Pro Besuch	364,40	KBV, EBM, Quartal 1, S. 555 [33]	2023
Ambulante therapeutische Behandlung	Ergotherapeut:in	1 h	73,41	GKV Spitzenverband, Vergütungsvereinbarung Anlage 2, S. 3, X4105 [59]	2021
	Physiotherapeut:in	20–30 min	19,03	Vdek, Gebührenübersicht für Therapeuten – Physiotherapie [60]	2021
	Neurolog:in	Bis zu 10 min	21,11	KBV, EBM, 2. Quartal, S. 401 [32] Mittelwert der altersspezifischen Angaben	2022
	Ostheopath:in	40–50 min	83,00	Verband der Osteopathen Deutschland e. V., Kosten: je 60–150 [61]	2023
	Homöopath:in	Pro Besuch	118,90	Gebührenordnung für Ärzte, Gesetze im Netz [62]	2023
	Akupunkteur:in	20 min	19,08	KBV, EBM, Quartal 2, S. 550 [34]	2023
	Heilpraktiker:in	30 min	12,30	Fachverband Deutscher Heilpraktiker [63]	2002
	Logopäd:in	30 min	46,12	GKV Spitzenverband, Vergütungsvereinbarung für Stimm‑, Sprech‑, Sprach- und Schlucktherapie [64]	2022
	Endokrinolog:in	1 Besuch	23,66	KBV, Quartal 2, EBM, S. 339 [32]	2022
	Ernährungsberater:in	1 h	80,00	Conze Ernährungsberaterin [65]	2018
	Sozialpädagogische Familienhilfe/Sozialarbeiter:in	1 h	20,18	Tarif öffentlicher Dienst [66]	2022
	Soziotherapie	1 h	20,18	Tarif öffentlicher Dienst [66]	2022
	Beratung (durch kirchliche oder kommunale Dienste, für Kinderberatung, Paare, sexuellen Missbrauch, Drogenmissbrauch …)	1 h	70,00	Hohberg [67]	2023
	Sozialpsychiatrische/r Beratung/Dienst (inkl. PIA)	1 h	95,89	KBV, EBM, Quartal 1, S. 479 (8,90 + (5*17,35); [31])	2022
	Psychiatrisches Gutachten	Pro Gutachten	80,00	Justizvergütungs- und -entschädigungsgesetz [68]	2021
	Religionsausübende (Priester, Imam, Rabbiner usw.)	1 h	26,80	Pfarrerbesoldungsgesetz [69]	2021
	(Chronische) Schmerztherapie	Pro Sitzung	45,28	KBV, EBM, Quartal 1, S. 542 [33]	2023

*InEK GmbH* Institut für das Entgeltsystem im Krankenhaus, *KBV* Kassenärztliche Bundesvereinigung, *EBM* Einheitlicher Bewertungsmaßstab, *KZVLB* Kassenzahnärztliche Vereinigung Land Brandenburg, *BEMA* Einheitlicher Bewertungsmaßstab für zahnärztliche Leistungen, *GKV* Gesetzliche Krankenversicherung, *Vdek* Verband der Ersatzkassen e. V., *PIA* Psychotherapeut:in in Ausbildung

### Statistische Analysen

Häufigkeiten der Inanspruchnahme wurden je Leistungseinheit aufaddiert. Fehlende Angaben wurden als „nicht in Anspruch genommen“ gewertet. Kosten wurden deskriptiv mit einem arithmetischen Mittelwert, Standardabweichung (Standard Deviation, SD) sowie dem minimalen und maximalen Wertebereich beschrieben.

Zur Berücksichtigung der Verteilungsschiefe der Kostenvariable wurde eine Kostenfunktion mit einem verallgemeinerten linearen Regressionsmodell (GLM) mit logistischer Linkfunktion und Gammaverteilung für Standardfehler ermittelt [38–40]. Die 6‑Monats-Gesamtkostenvariable diente als abhängige Variable (AV), die Variablen Alter, Gender (männlich/nicht männlich), Familienstand (verheiratet oder zusammenlebend mit Partner:in/allein lebend), Schichtdienst (Nein/Ja), Führungsverantwortung (Nein/Ja) und in Gruppen kodierte Diagnosen (keine Diagnose/Diagnosen) als unabhängige Variablen (UV). Das Signifikanzniveau wurde auf ein Alpha-Fehler-Niveau von *p* < 0,05 (2-seitig) festgelegt. Alle Analysen wurden mit IBM SPSS Statistics oder Stata 16 durchgeführt.

## Ergebnisse

An der Studie nahmen insgesamt 550 Arbeitnehmer:innen teil (301 Frauen, 247 Männer, 1 Person divers, 1 Person ohne Angabe). Das Durchschnittsalter betrug 46 Jahre (SD = 11), 39 % der Teilnehmenden waren alleinstehend und 60 % verheiratet oder in einer Partnerschaft lebend. Bei der Hälfte aller Teilnehmenden wurde im Rahmen des diagnostischen Interviews eine Depression diagnostiziert (*n* = 278). Bei insgesamt 84 (15 %) Befragten lag keine ICD-10-Diagnose vor, allerdings Symptome einer psychosomatischen Erkrankung (Tab. 2).

**Tab. 2:** Studienpopulation

Baseline		Insgesamt
		*N* = 550
*Alter*	M (SD)	46 (11)
*Gender*	Weiblich *n* (%)	301 (54,7)
	Divers *n* (%)	1 (0,20)
*Verheiratet/mit Partner:in lebend*	*n* (%)	332 (60)
*Im Schichtdienst tätig*	*n* (%)	74 (13,5)
*Mit Führungsverantwortung*	*n* (%)	116 (21)
*Krankheitsbedingte Fehltage*		
Gesamt (*N* = 550)	m (sd)	21,43 (29,80)
Personen mit Fehltagen (*n* = 409)		28,77 (31,32)
*GAF*	M (SD)	66,2 (7,6)
*Diagnosen (nach ICD-10)*		
Keine Diagnose^a^	*n* (%)	84 (15,3)
Depressionen (F32–33)		278 (50,5)
Angststörungen (F40–41)		40 (7,3)
Anpassungsstörungen (F43)		87 (15,8)
Somatoforme Störungen (F45)		19 (3,5)
Verhaltensauffälligkeiten mit körperlichen Störungen (F50–59)		11 (2)
Andere Diagnosen		28 (5)
Fehlend		3 (0,5)

*GAF* Global-Assessment-of-Functioning-Skala, *M* Mittelwert, *SD* Standardabweichung

^a^Symptome einer psychosomatischen Erkrankung ohne ICD-10-Diagnose gemessen mit dem GAF (Wert < 81)

Studienteilnehmende berichteten ein breites Spektrum an in Anspruch genommener Gesundheitsleistungen (Tab. 3). Im Rahmen einer ambulanten ärztlichen Behandlung wurden Hausärzt:innen (*n* = 400 Besuche, durchschnittliche Kontakte je Proband:in (Ø) 2,25–2,89) am häufigsten aufgesucht, gefolgt von sonstigen Fachärzt:innen (*n* = 206 Besuche, Ø 0,15–0,22) und Orthopäd:innen (*n* = 141 Besuche, Ø 0,65–0,69). Im Rahmen der ambulanten therapeutischen Behandlungen wurden Physiotherapeut:innen am häufigsten besucht (*n* = 65 Besuche, Ø 0,76–1,36), gefolgt von psychologischen Psychotherapeut:innen (*n* = 58 Besuche, Ø 0,15–0,45). Insgesamt nahmen Personen mit einer psychiatrischen Diagnose die meisten der gelisteten Leistungen etwas häufiger in Anspruch als Personen ohne psychiatrische Diagnose. Beispielsweise gaben 339 Personen (73 %, Ø 2,89) mit Diagnose an, in den letzten 3 Monaten bei einer/einem Hausärzt:in in Behandlung gewesen zu sein, im Vergleich zu 61 Personen (69 %, Ø 2,25) ohne Diagnose.

**Tab. 3:** Häufigkeit der Inanspruchnahme von Leistungen sowie durchschnittliche Kontakte je Proband:in vor Studienbeginn

Stationäre Krankenhausbehandlung	Häufigkeit	Keine Diagnose^a^/durchschnittliche Kontakte je Proband:in	Mit Diagnose^a^/durchschnittliche Kontakte je Proband:in
		M (SD)	M (SD)
Psychiatrische Klinik	1	1/1,14 (10,66)	0/−
Psychosomatische Klinik	7	0/−	7/0,20 (1,83)
Psychiatrische Rehabilitationsklinik	1	0/−	1/0,11 (2,33)
Psychosomatische Rehabilitationsklinik	7	1/0,41 (3,84)	6/0,47 (4,11)
Sonstige Krankenhäuser	34	6/0,11 (0,48)	28/0,14 (0,78)
*Behandlung in einer Tagesklinik*	*Häufigkeit*	*Keine Diagnose*	*Mit Diagnose*
Psychiatrische Tagesklinik	3	0/−	3/0,46 (6,45)
Psychosomatische Tagesklinik	2	1/0,20 (1,92)	1/0,12 (1,76)
Ambulante psychiatrische Rehabilitationsklinik	1	0/−	1/0,10 (2,23)
Ambulante psychosomatische Rehabilitationsklinik	0	0/−	0/−
Sonstige tagesklinische Einrichtungen	6	1/0,12 (1,12)	5/0,03 (0,45)
*Ambulante Ärztliche Behandlung*	*Häufigkeit*	*Keine Diagnose*	*Mit Diagnose*
Hausärztin/Hausarzt	400	61/2,25 (2,15)	339/2,89 (3,04)
*Ärztin/Arzt *für Psychiatrie	58	8/0,24 (1,02)	50/0,30 (0,99)
Ärztin/Arzt für psychosomatische Medizin	24	3/0,08 (0,55)	21/0,23 (2,02)
Internist:in	57	4/0,08 (0,46)	53/0,29 (1,11)
Orthopäd:in	141	24/0,65 (1,41)	117/0,69 (1,83)
Neurolog:in	66	10/0,14 (0,41)	56/0,23 (0,75)
Ärztin/Arzt für Arbeitsmedizin	73	7/0,10 (0,37)	66/0,21 (0,60)
Onkolog:in	5	0/−	5/0,02 (0,25)
Hautärztin/Hautarzt	106	18/0,25 (0,55)	88/0,31 (0,88)
Ärztin/Arzt für Hals-Nasen-Ohren-Heilkunde	87	11/0,20 (0,66)	76/0,29 (0,88)
Ärztin/Arzt für Augenheilkunde	77	17/0,64 (3,75)	60/0,18 (0,60)
Gynäkolog:in	129	22/0,33 (0,69)	107/0,35 (0,82)
Sonstige/r Fachärztin/Facharzt	206	47/0,22 (0,37)	159/0,15 (0,42)
*Klinikambulanz/ärztlicher Notdienst*	*Häufigkeit*	*Keine Diagnose*	*Mit Diagnose*
Psychiatrische Ambulanz	4	2/0,02 (0,15)	2/0,01 (0,24)
Psychosomatische Ambulanz	6	1/0,02 (0,21)	5/0,02 (0,19)
Notfallambulanz eines Allgemeinkrankenhauses	44	5/0,07 (0,30)	39/0,11 (0,40)
Ärztlicher Notdienst	16	3/0,03 (0,18)	13/0,03 (0,17)
Sonstige ambulante Klinik- oder Notarzttermine	13	4/0,02 (0,11)	9/0,02 (0,27)
*Ambulante therapeutische Behandlung*	*Häufigkeit*	*Keine Diagnose*	*Mit Diagnose*
Psychologische Psychotherapeut:in	58	6/0,15 (0,70)	52/0,45 (1,61)
Physiotherapeut:in	65	7/0,76 (4,29)	58/1,36 (5,00)
Ergotherapeut:in	4	0/−	4/0,15 (2,30)
Soziotherapeut:in	4	2/0,02 (0,15)	2/0,04 (0,70)
Homöopath:in	18	5/0,14 (0,59)	13/0,08 (0,61)
Akupunkteur:in	16	1/0,01 (0,11)	15/0,18 (1,20)
Praxis für Traditionelle Chinesische Medizin (TCM)	5	2/0,03 (0,24)	3/0,03 (0,43)
Naturheiler:in	14	5/0,14 (0,65)	9/0,05 (0,49)
Gesundbeter:in	1	0/−	1/0,01 (0,14)
Familientherapeut:in	3	0/−	3/0,02 (0,25)
Ernährungsberater:in	4	1/0,01 (0,11)	3/0,03 (0,40)
Sonstige Therapeut:in	32	7/0,11 (0,46)	25/0,19 (1,13)
*Onlinebehandlung oder -beratung*	*Häufigkeit*	*Keine Diagnose*	*Mit Diagnose*
Digitale ärztliche Sprechstunde	2	0/−	2/0,01 (0,19)
Digitale psychotherapeutische Sprechstunde	8	0/−	8/0,05 (0,41)
Sonstige digitale Angebote	6	3/0,09 (0,70)	3/0,06 (0,79)
*Gesundheitsbezogene Beratungsstellen*	*Häufigkeit*	*Keine Diagnose*	*Mit Diagnose*
Familien‑, Erziehungsberatung	6	2/0,02 (0,15)	4/0,08 (1,40)
Drogen‑, Suchtberatung	1	0/−	1/0,00 (0,05)
Schuldnerberatung	0	0/−	0/−
Koordinierungsstelle für frühe Hilfen (KoKi)	1	0/−	1/0,00 (0,05)
Psychologische Beratungsstelle	21	3/0,05 (0,26)	18/0,07 (0,38)
Sozialpsychiatrischer Dienst	10	4/0,08 (0,56)	6/0,03 (0,26)
Telefonseelsorge	4	1/0,02 (0,21)	3/0,02 (0,33)
Telefonischer Krisendienst	2	0/−	2/0,03 (0,43)
Sonstige Beratungsstellen oder Angebote	20	4/0,16 (1,01)	16/0,06 (0,63)

^a^Auf Basis des Mini-International Neuropsychiatric Interview (M.I.N.I.) zum Zeitpunkt des Studieneinschlusses

Die durchschnittlichen Gesamtkosten der Gesamtstichprobe für eine stationäre Krankenhausbehandlung in den vergangenen 6 Monaten lagen bei € 634,57 pro Person (SD: € 2801,39), für die Behandlung in einer Tagesklinik bei € 163,56 (SD: € 1627,15), für eine ambulant-ärztliche Behandlung bei € 161,18 (SD: € 256,46) und für die Behandlung in einer Klinikambulanz bzw. durch den ärztlichen Notdienst bei € 23,89 (SD: € 94,19). Die durchschnittlichen Kosten für Medikamente lagen bei € 154,28 (SD: € 624,33) für die vergangenen 6 Monate. Die indirekten Kosten aufgrund von Arbeitsunfähigkeitszeiten, die im Durchschnitt in den vergangenen 6 Monaten verursacht wurden, lagen bei € 3833,13 (SD: € 5395,25). Die durchschnittlichen Kosten, die für Zuzahlungen im Rahmen von Krankenhausbehandlungen, ärztlichen Leistungen u. Ä. in den letzten 6 Monaten zustande kamen, lagen bei € 106,42 (SD: € 216,25).

Die durchschnittlichen Gesamtkosten der vergangenen 6 Monate betrugen für die Gesamtstichprobe vor Studienbeginn € 5227,12 (SD: € 7704,21, Tab. 4).

**Tab. 4:** 6‑Monats-Kostenübersicht (in Euro)

				Wertebereich	
Variable	Fälle	Mittelwert	SD	Min.	Max.
Stationäre Krankenhausbehandlung	550	634,57	2801,39	0	27.792
Behandlung in einer Tagesklinik	550	163,56	1627,15	0	22.719,96
Ambulante ärztliche Behandlung	550	161,18	256,46	0	4456,44
Klinikambulanz/ärztlicher Notdienst	550	23,89	94,19	0	1564
Ambulante therapeutische Behandlung	550	112,18	338,04	0	4456,20
Onlineberatung	550	20,97	281,44	0	6300
Gesundheitsbezogene Beratungsstellen	550	16,95	63,30	0	605,40
Zuzahlungen	550	106,42	216,25	0	1757
Medikamente	550	154,28	624,33	0	7932
Indirekte Kosten	550	3833,13	5395,25	0	26.169,38
Gesamtkosten	550	5227,12	7704,21	0	54.364,87

*SD* Standardabweichung

Das allgemeine lineare Modell (General Linear Model, GLM) (Tab. 5) zeigt signifikante Effekte für das Alter und für Personen mit den Diagnosen Depression, Verhaltensauffälligkeiten mit körperlichen Symptomen und anderen Diagnosen (z. B. hyperkinetische Störung, Zwangsstörung). Mit jedem Lebensjahr steigen die Gesamtkosten um 3 % (Odds Ratio [OR] = 1,03; *p* < 0,01). Im Vergleich zu Teilnehmenden ohne Diagnose generieren Teilnehmende mit Depressionen 62 % höhere Kosten (OR = 1,617; *p* = 0,005); bei Personen mit Verhaltensauffälligkeiten mit körperlichen Symptomen bzw. anderen Diagnosen steigen die Kosten um das ca. 2,5-Fache (OR = 2,55; *p* = 0,034 bzw. OR = 2,40; *p* = 0,004).

**Tab. 5:** Allgemeines lineares Modell der Gesamtstichprobe

Gesamtkosten (€, abhängige Variable (AV))	exp (Koeffizient)	Standardfehler	t‑Wert	*p*-Wert	95 % Konfidenzintervall	Signifikanzniveau
*Gender (männlich)*	0,935	0,114	−0,55	0,580	0,736–1,187	–
*Alter*	1,03	0,006	5,25	–	1,018–1,040	***
*Familienstand (verheiratet/zusammenlebend mit Partner:in)*	0,862	0,108	−1,19	0,234	0,674–1,101	–
*Keine Diagnose*	1	–	–	–	–	–
Anpassungsstörung	1,252	0,261	1,08	0,282	0,831–1,885	–
Angststörung	1,126	0,298	0,45	0,654	0,670–1,89	–
Somatoforme Störung	1,753	0,617	1,59	0,111	0,879–3,50	–
Depressionen	1,617	0,278	2,79	0,005	1,154–2,266	***
Verhaltensauffälligkeiten mit körperlichen Störungen	2,55	1,126	2,12	0,034	0,194–1,058	**
Andere Diagnosen	2,40	0,728	2,88	0,004	0,775–2,471	***
*Schichtdienst*	1,347	0,238	1,69	0,092	0,953–1,903	*
*Führungsverantwortung*	0,821	0,120	−1,34	–	0,617–1,094	–
*Konstante*	972,528	308,197	21,71	0	522,580–1809,884	***
*Mittelwert AV*	5237,503		SD AV 7721,904			
*Fallzahl*	546		Chi-Square 57,660			
*Prob* *>* *χ*^*2*^	0,000		Akaike Crit. (AIC) 10366,808			

Linkfunktion = logistisch; family = gamma

*** *p*<0,01, ** *p*<0,05, * *p*<0,1

Nichtsignifikante Effekte zeigen sich für Gender, Familienstand und Führungsverantwortung. Es lässt sich folgender Trend erkennen: Im Vergleich zu Personen ohne Schichtdienst (Referenz) generieren Personen mit Schichtdiensttätigkeit 35 % höhere Kosten (OR = 1,347; *p* = 0,092).

## Diskussion

Obwohl die Studienteilnehmenden anhand ihres Diagnosespektrums und eines Anteils subklinischer Fälle von 15 % eher der CMD-Gruppe zuzurechnen sind, nahmen sie vielseitige Gesundheitsleistungen in Anspruch. Die 6‑Monats-Kosten der Studiengruppe (€ 5227,12, SD: € 7704,21) sind im Vergleich zu einer kürzlich untersuchten klinischen Studienpopulation (6-Monats-Kosten der Interventionsgruppe € 6726,10, SD: € 11.269,91; TAU-Gruppe: 9511,96, SD: € 15.616,87) ähnlich hoch [41]. Diese Ergebnisse deuten darauf hin, dass Gesundheitsleistungen bereits bevor es zu einer psychiatrischen Diagnose kommt zunehmend in Anspruch genommen werden und der Übergang von subklinischem zu klinischem Erkrankungsstadium fließend verläuft. Dies ist auffällig, da davon auszugehen ist, dass Personen, die bereits im klinischen System vorstellig wurden, einen höheren Leidensdruck aufweisen und somit mehr Leistungen in Anspruch nehmen. Es bleibt zu klären, welche Gründe es dafür geben könnte und ob diese Auffälligkeit auch im Längsschnitt bestehen bleibt.

Bereits vor Interventionsbeginn (Baseline) weisen Personen unserer Stichprobe, die Arbeitsunfähigkeitstage angegeben haben, eine mittlere Tageszahl von 29 Tagen auf. Dieser Wert entspricht der durchschnittlichen Falldauer psychischer Erkrankungen (29,6 Tage) für das Jahr 2022 laut Fehlzeitenreport 2023 [42]. Diesbezüglich erscheint unsere Stichprobe repräsentativ für Deutschland.

Mit jedem zusätzlichen Lebensjahr generieren Studienteilnehmende signifikant höhere Kosten im Gesundheits- und Sozialsystem. Der Einfluss des Alters auf die Inanspruchnahme von Gesundheitsleistungen ist insbesondere aus Studien zur somatischen Gesundheit bekannt, wurde allerdings auch für Personen mit psychischen Erkrankungen festgestellt [43, 44]. Insbesondere Studien in Populationen mit schweren psychischen Erkrankungen, wie z. B. Schizophrenie, zeigen demgegenüber häufiger negative Alterseffekte [45].

In der vorliegenden Analyse weisen Personen mit Führungsverantwortung niedrigere Kosten im Gesundheits- und Sozialsystem auf als Personen ohne Führungsverantwortung. Dieses Ergebnis ist aber nicht signifikant. Grund für niedrigere Inanspruchnahmen könnte die Sorge vor möglicher Stigmatisierung bei Inanspruchnahme solcher Leistungen sein. In der systematischen Übersichtsarbeit von Clement et al. wurde Stigma in allen Bevölkerungsgruppen als vierthäufigste Barriere für Hilfesuchverhalten genannt [46]. Ein weiterer Grund könnte eine höher wahrgenommene Anerkennung bei stärkerer Verausgabung nach dem Engagement-reward-Modell von Sigrist sein [47].

Die errechneten Kosten weisen einen sehr hohen Anteil (73 %) indirekter Kosten – auf Basis von Fehltagen – auf. Im nationalen Vergleich [41] liegt dieser Anteil je nach Studienpopulation bei bis zu 62 %. Auch im internationalen Vergleich ist dieser Anteil mit 33–40 % hoch [48–50]. Der Anteil von 73 % in der vorliegenden Studie ist nicht verwunderlich: Bei der Studienpopulation handelt es sich um Arbeitnehmer:innen, die anstelle von stationären Leistungen mehr ambulante Leistungen in Anspruch genommen haben (Tab. 3), aber ähnlich hohe Arbeitsunfähigkeitszeiten (28,77 Tage) wie die Allgemeinbevölkerung (29,6 Tage, [42]) aufweisen.

Gleichzeitig zeigt Bombana [51], dass bei klinischen Stichproben die stationären klinischen Kosten den höchsten Anteil der Gesamtkosten verursachen, der Anteil der indirekten Kosten verursacht einen geringeren Anteil. Unsere Ergebnisse zeigen signifikante Kostenunterschiede zwischen Personen mit bestimmten diagnostizierten psychischen Erkrankungen (Depressionen, Verhaltensauffälligkeiten mit körperlichen Symptomen und anderen Diagnosen) und Personen ohne Diagnose. Aus der Literatur ist bekannt, dass in Deutschland Personen mit Angsterkrankungen und Depressionen ca. 2‑ bis 2,5-fach höhere Kosten verursachen als Personen ohne die jeweilige psychische Erkrankung [52, 53]. In unserer Stichprobe konnten wir nur statistisch signifikant höhere Kosten (um 62 %) für Personen mit Depressionen zeigen, nicht aber für Personen mit Angsterkrankungen.

Für Personen mit Verhaltensauffälligkeiten mit körperlichen Symptomen (F50–59), wie z. B. Essstörungen, werden in einer Literaturanalyse jährliche Kosten von 1288–8042 US-Dollar pro Person genannt [54]. Die direkten Kosten für Personen mit Reizdarmsyndrom aus dem Jahr 2002 sind daher etwas veraltet und belaufen sich auf € 791 [55]. In dem systematischen Review von Schnorbach und Kruis [56] aus dem Jahr 2020 werden Gesamtkosten von € 6485 pro Person und Jahr angegeben. Allerdings sind die zuvor genannten Studien nicht im Arbeitskontext verortet oder beziehen sich nicht auf Deutschland [54–57]. So sind unsere Ergebnisse mit 2,5-mal höheren Kosten für Personen mit Verhaltensauffälligkeiten mit körperlichen Symptomen im Vergleich zu Personen ohne Diagnose schwer einzuordnen.

Folgende Limitationen sind zu nennen: Die Form der Ansprache möglicher Studienteilnehmenden im Betrieb kann zu einer gewissen Selbstselektion geführt haben. Zudem führten die verschiedenen Rekrutierungswege zu einer gewissen Heterogenität innerhalb der Stichprobe, sodass diese als nicht repräsentativ für alle Arbeitnehmer:innen betrachtet werden kann. Des Weiteren ist zu nennen, dass Personen evtl. nicht alle Termine oder Leistungen im Gesundheitssystem erinnern. Kosten werden daher möglicherweise unterschätzt. Gleichzeitig wird ein Teil der Kosten auf 6 Monate hochgerechnet, was zu einer Überschätzung der Kosten führen könnte. Das Fehlen aktueller standardisierter Kostensätze in Deutschland limitiert zudem die Vergleichbarkeit unserer Ergebnisse mit Ergebnissen anderer Studien, die andere Messzeitpunkte und Kostensätze nutzen.

## Fazit

Die Ergebnisse der Untersuchung liefern Hinweise darauf, dass die Inanspruchnahme und die Kosten von Gesundheitsleistungen in einer Stichprobe von berufstätigen Personen mit dem Vorliegen der Kriterien für bestimmte psychiatrische Diagnosen sowie dem Alter im Zusammenhang stehen. Weitere Untersuchungen dazu, ob dieses Phänomen auch im Längsschnitt Bestand hat, sind notwendig.
